# Comparison of Superhydrophilic, Liquid-Like, Liquid-Infused, and Superhydrophobic Surfaces in Preventing Catheter-Associated Urinary Tract Infection and Encrustation

**DOI:** 10.1021/acsbiomaterials.3c01577

**Published:** 2024-01-06

**Authors:** Xiao Teng, Chenghao Yao, Colin P. McCoy, Shuai Zhang

**Affiliations:** School of Pharmacy, Queen’s University Belfast, Belfast BT9 7BL, U.K.

**Keywords:** urinary catheter, coating, biofilm, encrustation, infection, migration

## Abstract

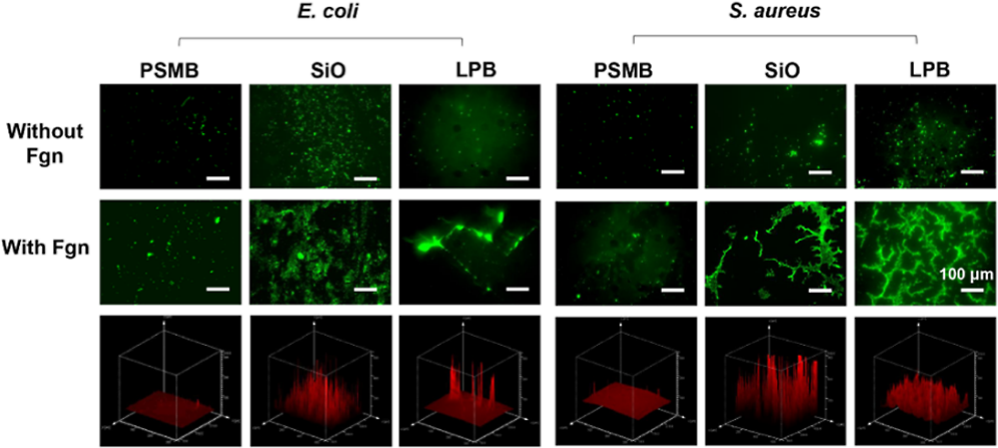

Over the past decade, superhydrophilic zwitterionic surfaces, slippery liquid-infused porous surfaces, covalently attached liquid-like surfaces, and superhydrophobic surfaces have emerged as the most promising strategies to prevent biofouling on biomedical devices. Despite working through different mechanisms, they have demonstrated superior efficacy in preventing the adhesion of biomolecules (e.g., proteins and bacteria) compared with conventional material surfaces. However, their potential in combating catheter-associated urinary tract infection (CAUTI) remains uncertain. In this research, we present the fabrication of these four coatings for urinary catheters and conduct a comparative assessment of their antifouling properties through a stepwise approach. Notably, the superhydrophilic zwitterionic coating demonstrated the highest antifouling activity, reducing 72.3% of fibrinogen deposition and over 75% of bacterial adhesion (*Escherichia coli* and *Staphylococcus aureus*) when compared with an uncoated polyvinyl chloride (PVC) surface. The zwitterionic coating also exhibited robust repellence against blood and improved surface lubricity, decreasing the dynamic coefficient of friction from 0.63 to 0.35 as compared with the PVC surface. Despite the fact that the superhydrophilic zwitterionic and hydrophobic liquid-like surfaces showed great promise in retarding crystalline biofilm formation in the presence of *Proteus mirabilis*, it is worth noting that their long-term antifouling efficacy may be compromised by the proliferation and migration of colonized bacteria as they are unable to kill them or inhibit their swarming. These findings underscore both the potential and limitations of these ultralow fouling materials as urinary catheter coatings for preventing CAUTI.

## Introduction

Biofilm formation and encrustation remain the two main issues afflicting urinary catheters to date.^1^ Despite exhibiting variations in their underlying mechanisms, they can often overlap and make conditions worse in an infection, making it extremely difficult to prevent.^2^ Catheter-associated urinary tract infection (CAUTI) usually begins with bacterial colonization on catheter surfaces, followed by their ascent to the bladder lumen and dissemination into the kidneys and other organs.^3^ Certain urease-producing bacteria can also hydrolyze urea and raise urinary pH, leading to catheter encrustation by crystalline deposits that can block urine flow and form “infection stones” in the bladder, causing severe complications (e.g., pyelonephritis and septicemia) and an increased rate of morbidity and mortality.^4,5^ Over the past few decades, commercial anti-infection catheters have mainly focused on impregnating or depositing antibiotics (e.g., nitrofurazone) or silver onto the catheter surfaces.^2,6,7^ However, these catheters have been disappointing in clinical use and have increased the risk of developing antimicrobial resistance in bacteria.^8^

As CAUTI begins with bacterial adherence to a catheter surface, an alternative strategy has focused on inhibiting bacterial colonization by controlling bacteria–surface interactions.^9^ Baier first demonstrated the correlation between the relative adhesion of bacteria and surface energy and found that there exists optimum energy for the surface to maximumly reduce bacterial attachment in an aqueous condition.^10^ This optimum surface energy (∼20 to 25 mJ/m^2^) is approximately equal to the dispersive component for water (∼21.8 mJ/m^2^), which allows water to rewet the surface and remove attached bacteria at a minimum “thermodynamic cost”. However, in a complex physiological environment, the first change to a catheter surface is often the deposition of a complex set of host-generated proteins and biological molecules (conditioning film), which can mask the surface before bacterial colonization.^11^ For example, urinary catheterization induces mechanical stress that may cause histological and immunological changes in the bladder, resulting in a robust inflammatory response and triggering the release of fibrinogen (Fgn).^12^ Fgn can readily adsorb to the catheter surface and promote bacterial binding, thereby accelerating biofilm formation and potentiating infections.^13^ Therefore, a catheter surface capable of inhibiting protein adsorption and biofilm formation would be essential to preventing or retarding CAUTI.

According to the classic Derjaguin–Landau–Verwey–Overbeek theory, the binding of biofouling (bacteria and proteins) to a solid surface is governed by a range of physio–chemical interactions.^14^ Our recent studies have shown that there exist two optimum surface energies for minimum adhesion of bacteria (∼25 mJ/m^2^) and proteins (∼35 mJ/m^2^), respectively.^13,15^ The surface energies of conventional catheter materials and coatings [e.g., silicone, polyvinyl chloride (PVC), hydrogels, and silver] typically fall outside the range of their use, making it impossible to simultaneously repel bacteria and proteins. On the other hand, the recent emergence of biocompatible ultralow-fouling surfaces, including superhydrophilic zwitterionic surfaces,^16^ slippery liquid-infused porous surfaces,^17−19^ covalently attached liquid-like surfaces,^20^ and superhydrophobic (SH) surfaces,^21^ have garnered significant attention and shown great promise in preventing biofouling on medical devices.^22,23^ Despite working through different mechanisms, these surfaces can form a “dynamic interface” between the surface and foulants (e.g., bacteria and proteins), inhibiting their attachment and propagation or allowing easy detachment under shear flow.^20^ To date, numerous studies have reported their success under various conditions, but no research has been conducted to compare their efficacy in preventing CAUTI.^24−26^ Herein, we describe the fabrication of these four types of coatings for urinary catheters and compare their antifouling performance with that of uncoated and hydrogel-coated catheters using a stepwise approach. Their surface properties, including surface morphologies, chemical compositions, wettability, surface energy, and friction coefficient, were also characterized and compared.

## Materials and Methods

### Materials

Medical-grade unplasticized PVC sheets were purchased from Goodfellow Cambridge Ltd. (Huntingdon, UK). Acetic acid glacial was purchased from VWR Chemicals (Lutterworth, UK). The SYLGARD 184 elastomer kit was purchased from Dow Corning Corporation (Midland, UK). Kollidon 90F was purchased from BASF (Ludwigshafen, Germany). *Escherichia coli* (*E. coli*, ATCC 25922) and *Staphylococcus aureus* (ATCC 29213) were obtained from the American Type Culture Collection (ATCC, Buckinghamshire, UK). The LIVE/DEAD BacLight Bacterial Viability Kit L13152 was purchased from Thermo Fisher Scientific (Paisley, UK). Other chemicals used in this study were purchased from Merck Life Science UK Ltd. (Dorset, UK) without further purification.

### Coating Fabrication

Poly(sulfobetaine methacrylate) (PSBMA) was selected as a model material and coated onto the PVC substrate via photopolymerization for the zwitterionic coating. In brief, the PVC surface was ultrasonically etched (40 kHz) in ethanol for 5 min^27^ and immersed in the coating solution comprising 0.5 M SBMA, 1 mM *N*,*N*′-methylene-bis-acrylamide, and a suitable amount of 2-hydroxy-4′-(2-hydroxyethoxy)-2-methylpropiophenone, followed by UV irradiation (wavelength: 365 nm) for 30 min. The PSBMA-coated samples were then stored in deionized water before further use.

For the hydrogel coating (HC), poly(hydroxyethyl methacrylate-*co*-*N*-vinylpyrrolidone) (HEMA-*co*-NVP) hydrogel was used as a model material and coated onto the PVC substrate via a dip coating approach.^28^ The hydrogel precursor solution comprises 1 M 2-HEMA and NVP (1:1, mol/mol), 0.1 mM MBA, 0.2 mM 2-hydroxy-4′-(2-hydroxyethoxy)-2-methylpropiophenone, and 10% (w/v) Kollidon 90F. The PVC substrate was dipped into the solution and withdrawn at a constant speed of 50 mm/min at room temperature. After photopolymerization for 20 min, the samples were immersed in deionized water for 48 h to remove unreacted monomers.

The liquid-infused surface was fabricated by immersing silicone oil (SiO) into a polydimethylsiloxane (PDMS) matrix using the method detailed by Ozkan et al.^29^ In brief, a PDMS precursor solution was prepared by mixing a SYLGARD 184 elastomer kit and a curing agent (10:1, w/w) according to the manufacturer’s instructions. The solution was then poured into a Petri dish and degassed in a vacuum chamber for 1 h to eliminate bubbles. After being cured at 60 °C for 24 h, the PDMS disks were then cut into small sizes with a diameter of 1 cm and immersed in silicone oil (viscosity 10 cSt at 25 °C) for at least 12 h to allow the oil to infiltrate the polymer networks. The excess oil was removed by wiping with Kleenex tissues, and the samples were sterilized with ethanol before further use.

For the covalently bound liquid-like surface, a liquid-like PDMS brush (LPB) was selected as a model material and deposited on the PVC substrate using a modified method described by Armstrong et al.^30^ In brief, oxygen plasma-treated PVC sheets were immersed in a reactive solution of isopropanol, dimethyldimethoxysilane, and sulfuric acid (90, 9, and 1 wt %) for 10 s and then slowly withdrawn at room temperature. The samples were then stored in a homemade humidity chamber at 50–60% relative humidity. After 1 h, the samples were taken out, washed extensively with deionized water and isopropyl alcohol, and stored in a desiccator before further use.

The SH surface was created on PDMS sheets using the sol–gel method. First, hydrophobic TiO_2_ particles were prepared by mixing two different size ranges of TiO_2_ nanoparticles (∼25 and ∼100 nm) in ethanol with perfluorooctyltriethoxysilane (1%, v/v). After 12 h, the hydrophobic TiO_2_ particles were harvested and ultrasonically dispersed in the PDMS precursor solution [10% (v/v) in tetrahydrofuran] described above. The SH coating was then deposited on the PDMS substrate through a dip coating process, and the samples were dried at 60 °C overnight before use.

### Characterization

The surface morphologies of the coatings were characterized by using a dual-beam focused ion beam scanning electron microscope (TESCAN LYRA3, Brno, Czech Republic) with an accelerated voltage of 5 kV. For the SiO-infused surface, the accelerated voltage was set at 3 kV to increase image stability and resolution. To measure the coating thickness, the samples were frozen with liquid nitrogen and cut with a diamond cutter, and the cross-sectional areas were observed by microscopy. The chemical compositions of the coatings were characterized using attenuated total reflection-Fourier transform infrared (ATR–FTIR) spectroscopy (PIKE MIRacle, Madison, USA). The surface wettability was determined by a sessile drop method using an optical tensiometer (Theta Flow, Bolin Scientific, Sweden). The surface energy and its components of the coatings were calculated using the Van Oss method.^31^ The advancing and receding contact angles of deionized water and Fgn solution (2.6 mg/mL) were measured while the probe fluid was added to and withdrawn from the drop. The static and dynamic coefficients of friction (COF) of the surface were determined by using a COF tester (COF-1000, USA) according to ASTM D1894.^32^

### Protein/Blood Adsorption Assay

Fgn from human plasma was diluted to 2.6 mg/mL in PBS and added to each sample in a 48-well plate. The plate was sealed with Parafilm and left at 4 °C. After 24 h, the samples were taken out and rinsed three times with PBS, followed by ultrasonication in sodium dodecyl sulfate for 15 min. The total amount of adsorbed Fgn was then determined using a bicinchoninic acid approach.^1^ To investigate the distribution of Fgn on the surface, the samples were treated with Alexa Fluor 488 conjugated Fgn under the same conditions, and the adsorbed proteins were visualized by fluorescent microscopy (Leica DM5500, Berlin, Germany). To further assess the surface repellence, the samples were exposed to sheep whole blood for 30 s and 1 h, respectively, and the blood coagulation on the surfaces was visualized and compared. According to Huang et al.,^33^ to quantify the platelet adhesion, the fresh sheep blood was centrifuged at 2000 rpm for 10 min to obtain platelet-rich plasma (PRP), and 100 μL of the PRP was dropped onto the surface of each sample and coincubated at 37 °C. After 1 h, the samples were carefully washed with PBS to remove nonadherent platelets and placed in 1% triton-X100 at 37 °C for 1 h to cleave adhered platelets. The relative level of adhered platelets was then quantified using a lactate dehydrogenase (LDH) kit, and the absorbance (OD) at 490 nm was detected by a microplate reader. 100 μL of PRP was set as the positive control, and the relative quantity of adhered platelets was expressed by the following formula: ODsample/ODpositive control.

### Biofilm Formation

The antibiofilm performance of the surfaces was investigated using a stepwise approach. First, the samples were challenged with neat *E. coli* or *S. aureus* suspension (∼2 × 10^8^ cfu/mL in PBS) at 37 °C for 24 h under static conditions and up to 72 h under dynamic flow conditions (flow rate: 0.75 mL min^–1^),^34^ respectively. The adhered cells were quantified using a plate count method. To investigate the effect of Fgn adsorption on bacterial binding, the samples were preconditioned with human Fgn for 24 h and incubated with bacteria at the same condition. The adhered biomass was stained and examined by fluorescent microscopy. To investigate their long-term antibiofilm efficacy, the surfaces were preconditioned with Fgn at the same condition and challenged with bacteria for 3 days. The bacteria suspension was refreshed daily, and the biofilm formed on the surface was observed and analyzed with the Leica Application Suite X 1.4.5 (Leica, Berlin, Germany).

### Encrustation Assay

The antiencrustation properties of coatings were examined using a modified encrustation model described by Jones et al.^35^ The Fgn-conditioned sample was perpendicularly immersed in 2 mL of *Proteus mirabilis* ATCC 51286 (*P. mirabilis*, ∼ 1 × 10^8^ cfu/mL) in artificial urine at 37 °C for 12 h. The pH change at 0, 3, 6, and 12 h was monitored. The samples were also taken out, dehydrated, and gold-coated for SEM imaging at each time point.

### Statistical Analysis

All data are presented as the mean ± the standard deviation. A one-way ANOVA (Tukey’s post hoc) was performed to determine statistical significance, where values of *p* < 0.05 were considered significant and *p* < 0.01 were considered highly significant.

## Results and Discussion

### Surface Characterization

**Figure 1:**
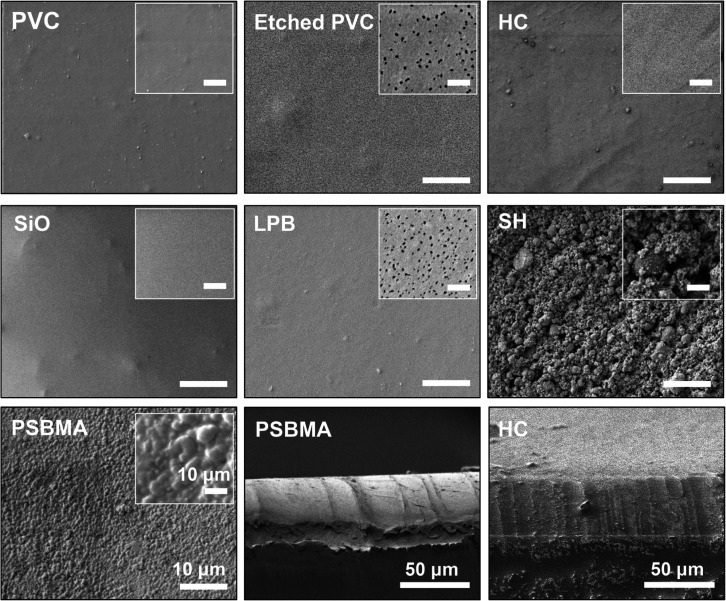
Typical top and cross-sectional SEM images of different surfaces (scale bars correspond to 10 and 50 μm, respectively).

**Figure 2:**
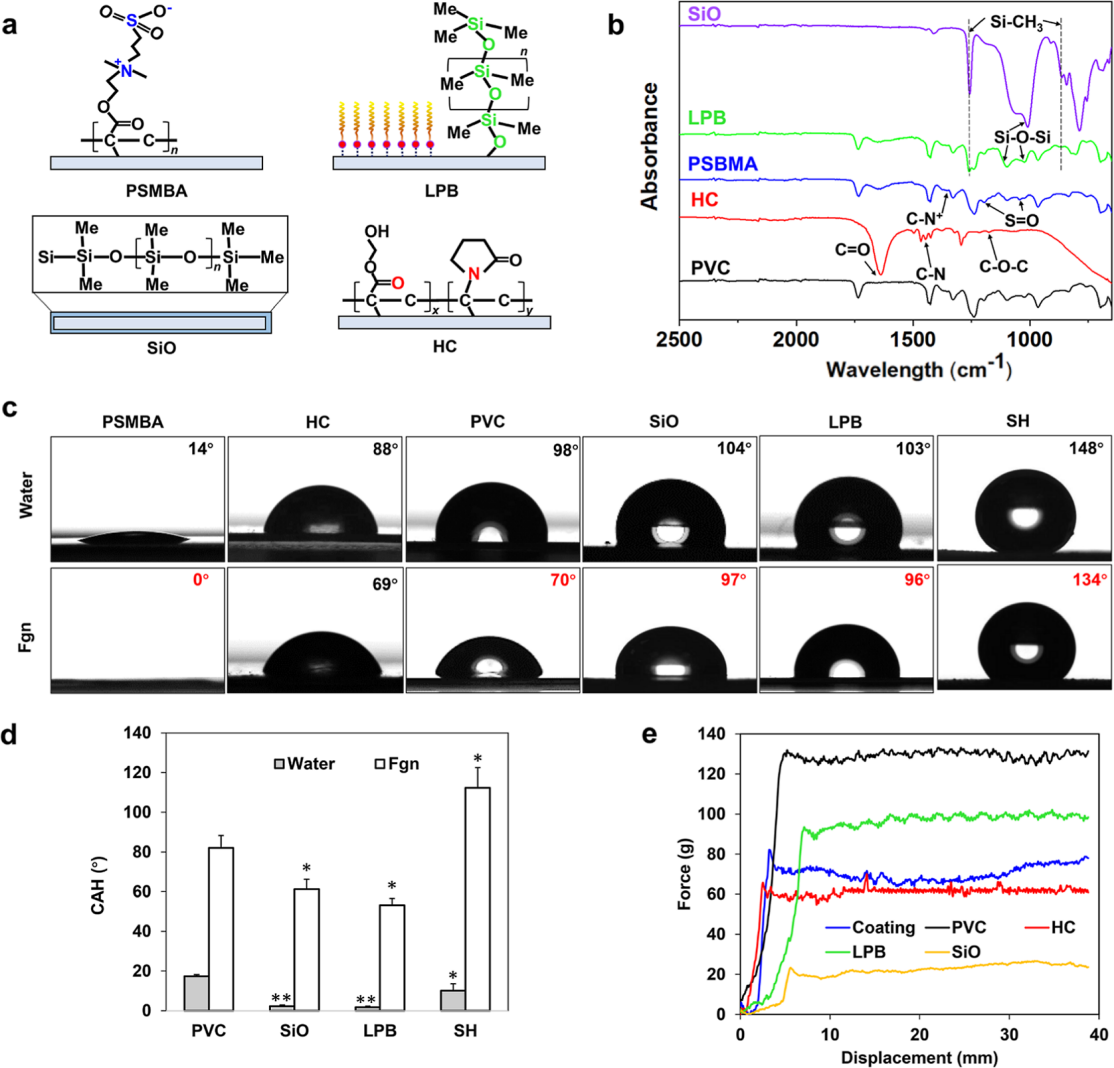
(a) Chemical structures of PSBMA, LPB, SiO, and HC; (b) ATR–FTIR spectra (Me stands for CH3); (c) contact angle profiles of water and Fgn on different surfaces; (d) comparison of the water and Fgn CAHs on different surfaces; and (e) typical friction test curves: friction force versus displacement (*n* = 6, bars stand for the standard deviation of the mean; *p < 0.05 and **p < 0.01 compared with PVC).

To enhance the bonding strength of the coatings, the PVC substrate was etched with ethanol to create nanopores (diameter: ∼50 to 100 nm) (Figure 1) to promote mechanical interlocking at the coating-substrate interface.^27^ After being coated with various materials; the surfaces displayed different morphologies and levels of roughness. Among the hydrophobic coatings, the SiO-infused PDMS exhibited the smoothest surface with an oil layer of ∼30 μm in thickness.^1^ The LPB coating also displayed a smooth surface, but it was reported that the thickness of LPB remained stable at only ∼8 to 10 nm,^36^ which was challenging to measure by SEM in this study. Although the plasma-etched PVC surface exhibited a higher pore density than the untreated PVC, the relatively small pore size (diameter: ∼5 to 10 nm) was unlikely to affect the overall grafting density and uniformity of the coating. This was evidenced by the low water contact angle hysteresis (CAH, 2.2 ± 0.4°), which was very close to that of the SiO-infused surface (1.4 ± 0.5°) (*p* > 0.05). As shown in Figure 2d, the water CAHs of SiO (*p* < 0.01), LPB (*p* < 0.01), and SH (*p* < 0.05) were significantly lower than those of PVC, and the SH showed a significantly higher water CAH than SiO and LPB (*p* < 0.05). Figure 2a illustrates the chemical structure of LPB, and its chemical composition was verified by FTIR spectroscopy (Figure 2b). Despite the variation in coating thickness, the SiO-infused and LPB-coated surfaces exhibited similar water repellence (Figure 2c) due to their similar chemical properties. The SH coating showed a typical rough surface with the aggregation of hydrophobic TiO_2_ nanoparticles forming a hierarchical micro/nano surface structure, entrapping air pockets, and making it super-repellent to water (WCA ∼ 150°).^37^

For the hydrophilic coatings, the HC coating was smoother and thicker (∼40 μm) than the PSBMA coating (∼8 μm) due to the presence of a thickening agent (PVP). Their chemical compositions were verified by FTIR spectroscopy (Figure 2b). The HC coating shows characteristic absorption peaks at 1638 cm^–1^ (C=O stretching), 1174 cm^–1^ (C–O–C bending), and 1496 cm^–1^ (C–N stretching), which are consistent with the results reported in other studies.^38^ The PSBMA coating also shows characteristic absorption bands at 1354 cm^–1^ (quaternary ammonium group), 1052, and 1196 cm^–1^ (symmetric and asymmetric S=O stretchings), respectively.^39^ Upon contact with water, the PSBMA coating became superhydrophilic within 1 min (WCA decreased from ∼14 to 8°), while the HC coating took a longer time to absorb water, swell, and become hydrophilic (WCA changed from ∼88 to ∼49° after 10 min). To ensure that the coatings were fully hydrated before the friction test, we immersed the samples in deionized water for 1 h to reach an equilibrium state (indicated by a WCA of 0°). As shown in Figure 2e, the hydrated HC coating exhibited enhanced lubricating properties when compared to the bare PVC surface, decreasing the static COF (SCOF) and dynamic COF (DCOF) from 0.63 to 0.34 (*p* < 0.05) and 0.61 to 0.27 (*p* < 0.05), respectively. The hydrated PSBMA coating also showed improved lubricity (SCOF 0.41 and DCOF 0.35) compared to the PVC surface (*p* < 0.05), but its COF values (SCOF 0.41 and DCOF 0.35) were higher than those of the HC coating (*p* < 0.05). This is because the thicker hydrogel layer can hold a higher level of water to provide a more resilient fluid interfacial layer during friction.^40^ The hydrophobic SiO-infused surface displayed the lowest surface friction (SCOF 0.23 and DCOF 0.21, *p* < 0.01 compared with PVC), but the friction coefficient increased rapidly after five repeating cycles due to oil depletion (exposed PDMS substrate observed by microscopy). In comparison, the covalently bound LPB coating remained stable even after 50 cycles, but its ultralow thickness led to relatively higher COF values (SCOF 0.46 and DCOF 0.43) compared to the HC coating (*p* < 0.05) and SiO-infused surface (*p* < 0.01). No significant difference was found in both SCOF and DCOF between LPB and PSBMA. The enhanced surface lubricity of HC, PSBMA, and LPB coatings may reduce the likelihood of physical damage to the bladder and urethra and convey additional anti-inflammatory and antibiofouling benefits.

### Protein/Blood Adsorption

**Figure 3:**
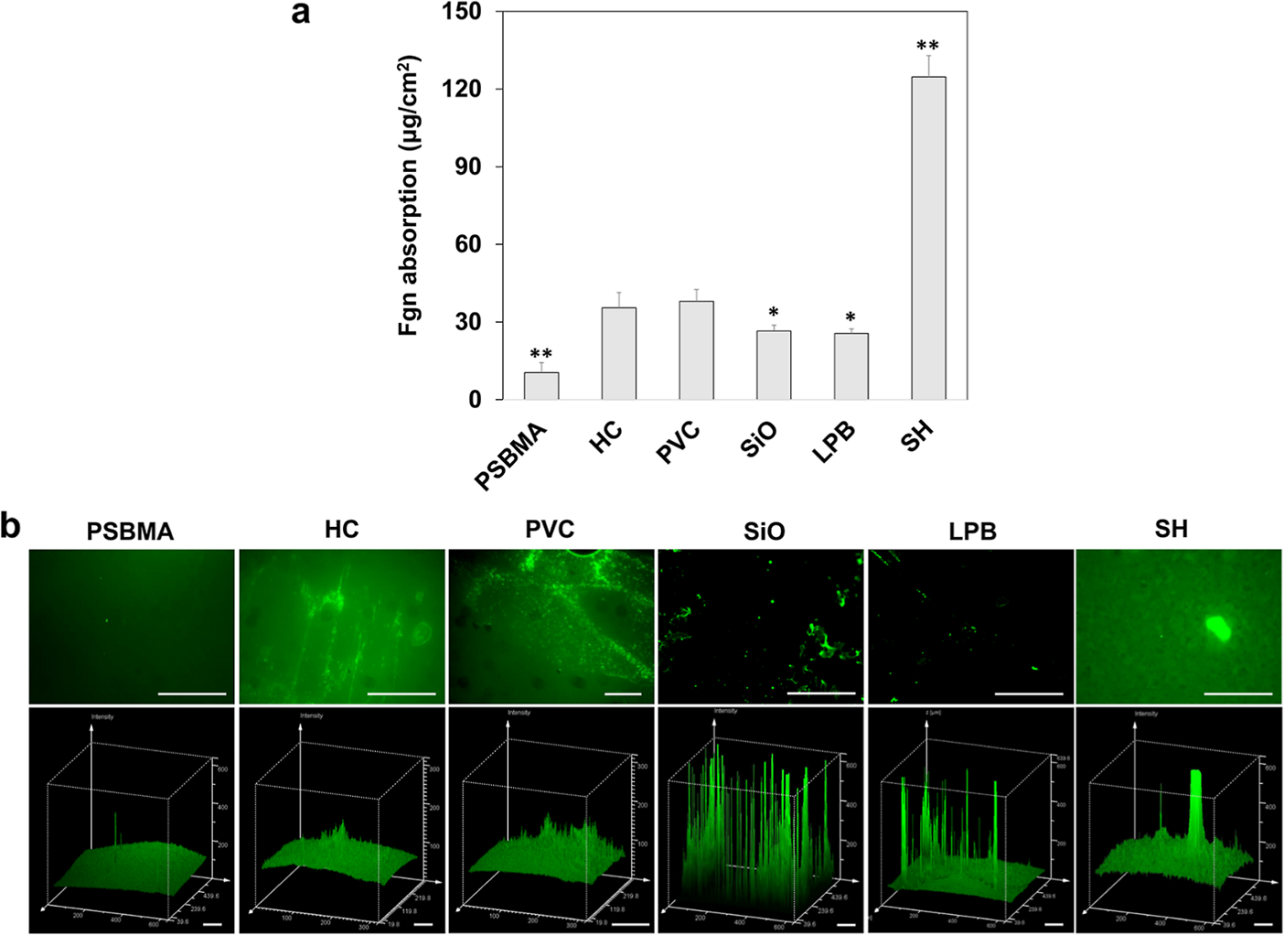
(a) Amount of Fgn adsorbed on different surfaces after 2 h of coincubation and (b) typical fluorescent images of different surfaces after Fgn adsorption (*n* = 12, bars stand for the standard deviation of the mean, scale bars correspond to 100 μm; *p < 0.05 and **p < 0.01 compared with PVC).

Considering the exploitative interaction of uropathogens with deposited Fgn, we hypothesized that a catheter surface capable of preventing Fgn adsorption would reduce the level of bacterial colonization and retard biofilm formation. As shown in Figure 3a, the PSBMA coating demonstrated the highest antiprotein activity, reducing Fgn adhesion by 72.3 and 70.4% compared to the bare PVC surface and HC coating (*p* < 0.01), respectively. Different from HEMA or other conventional hydrogel materials, which bind water via hydrogen bonding, zwitterionic materials hold water more strongly through ionic solvation.^41^ This enables the formation of a more stable and highly structured hydration layer on the PSBMA coating, allowing proteins to maintain a stable conformation when approaching the surface, thereby preventing irreversible adsorption.^42^ To verify this, we used fluorescently labeled Fgn to investigate its adsorption behavior under the same conditions. As seen in Figure 3b, the PSBMA coating was much more refractory to Fgn adsorption than other surfaces, as only a small amount of scattered proteins were observed on its surface, while significant Fgn aggregation was seen on the HC surface, even though it was fully hydrated prior to the test. The results indicate that the hydrophilic nature of HC was not enough to resist protein adhesion, and no significant difference was found compared to PVC (*p* > 0.05). The amphiphilic nature of these hydrogels (i.e., HEMA and PVP) allows them to selectively mask the hydrophobic domains of proteins in aqueous solutions, while the zwitterionic PSBMA avoids hydrophobic interactions due to its highly charged groups.^43^

**Table 1:** Contact Angle and Surface Energy of Different Surfaces (*n* = 6; Bars Stand for the Standard Deviation of the Mean)

	contact angle, θ (deg)			surface free energy (mJ/m^2^)			
	θ^W^	θ^D^	θ^E^	γ^LW^	γ^+^	γ^–^	γ^TOT^
PVC	97.5 ± 0.3	45.5 ± 4.1	73.5 ± 1.9	36.74	0.25	0.03	36.90
PSBMA	13.9 ± 2.8	35.5 ± 1.1	20.9 ± 2.9	41.80	0.00	67.08	42.41
HC	49.4 ± 3.8	53.8 ± 2.9	57.6 ± 2.4	32.13	0.28	37.57	38.61
SiO	107.4 ± 0.4	74.0 ± 3.8	86.0 ± 2.1	20.66	0.00	0.72	20.67
LPB	102.8 ± 2.1	65.0 ± 3.1	68.2 ± 3.9	25.70	0.63	0.02	25.91
SH	146.4 ± 3.3	89.5 ± 3.9	119.5 ± 2.0	12.92	0.60	1.81	15.01

For the SH coating, its antiprotein effectiveness strongly depends on the lifetime of the nonwetting (Cassie) state. Although the SH demonstrated outstanding water repellency (WCA ∼ 150°), it failed to resist Fgn adsorption under static conditions. Figure 2d shows a dramatic increase in CAH from ∼10 to ∼112° in the presence of Fgn. The lower surface tension of Fgn solution caused a wetting transition from the Cassie state to the Wenzel state (Figure 2c) and accelerated protein adsorption through stronger hydrophobic–hydrophobic interactions than the bare PVC (*p* < 0.01) (Figure 3a).^44^ In comparison, the SiO-infused and LPB-coated surfaces exhibited similar but significantly lower Fgn CAHs due to their smooth topographies (*p* < 0.05), suggesting a significant reduction in the force required to induce droplet shedding by motion along the surface.^12^ However, no significant difference in Fgn absorption was found between SiO and LPB (*p* > 0.05). It should be noted that we only compared the antiprotein efficacies of different surfaces under static conditions in this research, as this was to mimic the real condition in the bladder. Following bladder inflammation, Fgn is released into urine stored in the bladder and becomes adsorbed onto the catheter surface, where there is no urine flushing, similar to the inner lumen of the catheters. As shown in Figure 3a, the SiO and LPB reduced ∼30% of Fgn adhesion compared to the bare PVC, but the SiO-infused surface induced a significantly higher percentage of protein aggregation (Figure 3b). This is consistent with our recent finding that the SiO-Fgn interactions could lead to a conformational change in Fgn and cause protein denaturation over time.^1^ Instead, the LPB-coated surface consists of highly mobile but lower-molecular-weight PDMS chains that can prevent protein aggregation via dynamic motions such as stretching, bending, and rotating.^44^

**Figure 4:**
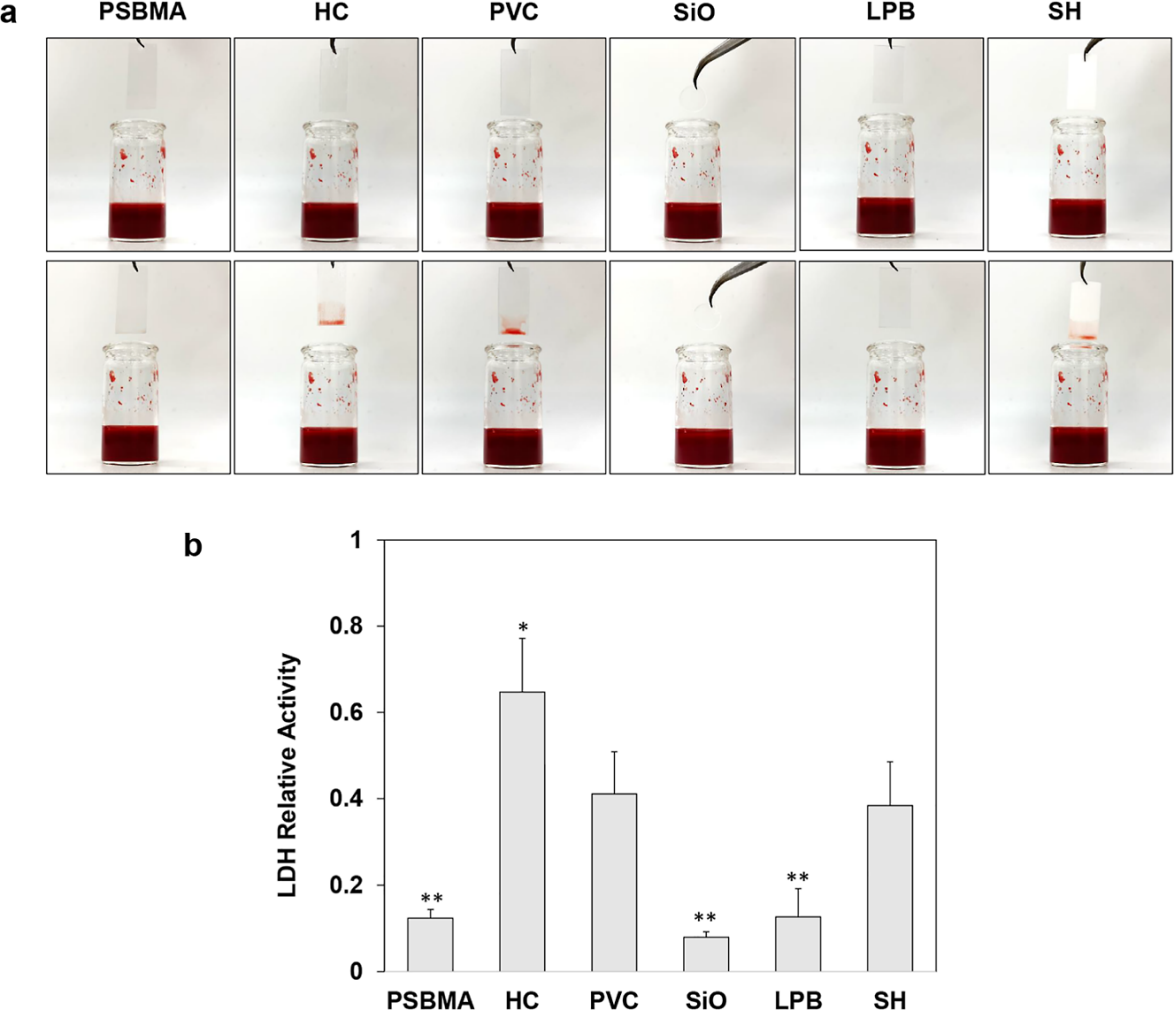
(a) Typical images of different surfaces before and after contact with whole sheep blood for 30 s and (b) LDH relative activities of different surfaces after contact with whole sheep blood for 1 h (*n* = 6, bars stand for the standard deviation of the mean; *p < 0.05 and **p < 0.01 compared with PVC).

On the other hand, repeated catheterization may cause urethral irritation and bleeding due to friction between the urethral mucosa and the catheter.^45^ This could trigger adverse events such as platelet adhesion and activation, and cause blood clot formation, block urine flow, and induce inflammation.^46^Figure 4a shows that the surfaces coated with PVC, HC, and SH displayed significant blood adhesion and retention, whereas the PSBMA, SiO-infused, and LPB-coated surfaces exhibited outstanding blood repellency, with blood droplets immediately slipping away without leaving any visible residue. The LDH results further supported the observation from a quantitative point of view: LDH relative activity was ∼0.41 in PVC while about 0.08 in SiO and 0.12 in PSBMA and LPB, respectively (Figure 4b). These results indicate that the zwitterionic PSBMA-coated, SiO-infused, and LPB-coated surfaces demonstrated superior resistance to proteins and blood, posing great potential to prevent biofouling on urinary catheters.

### Bacterial Adhesion and Biofilm Formation

**Figure 5:**
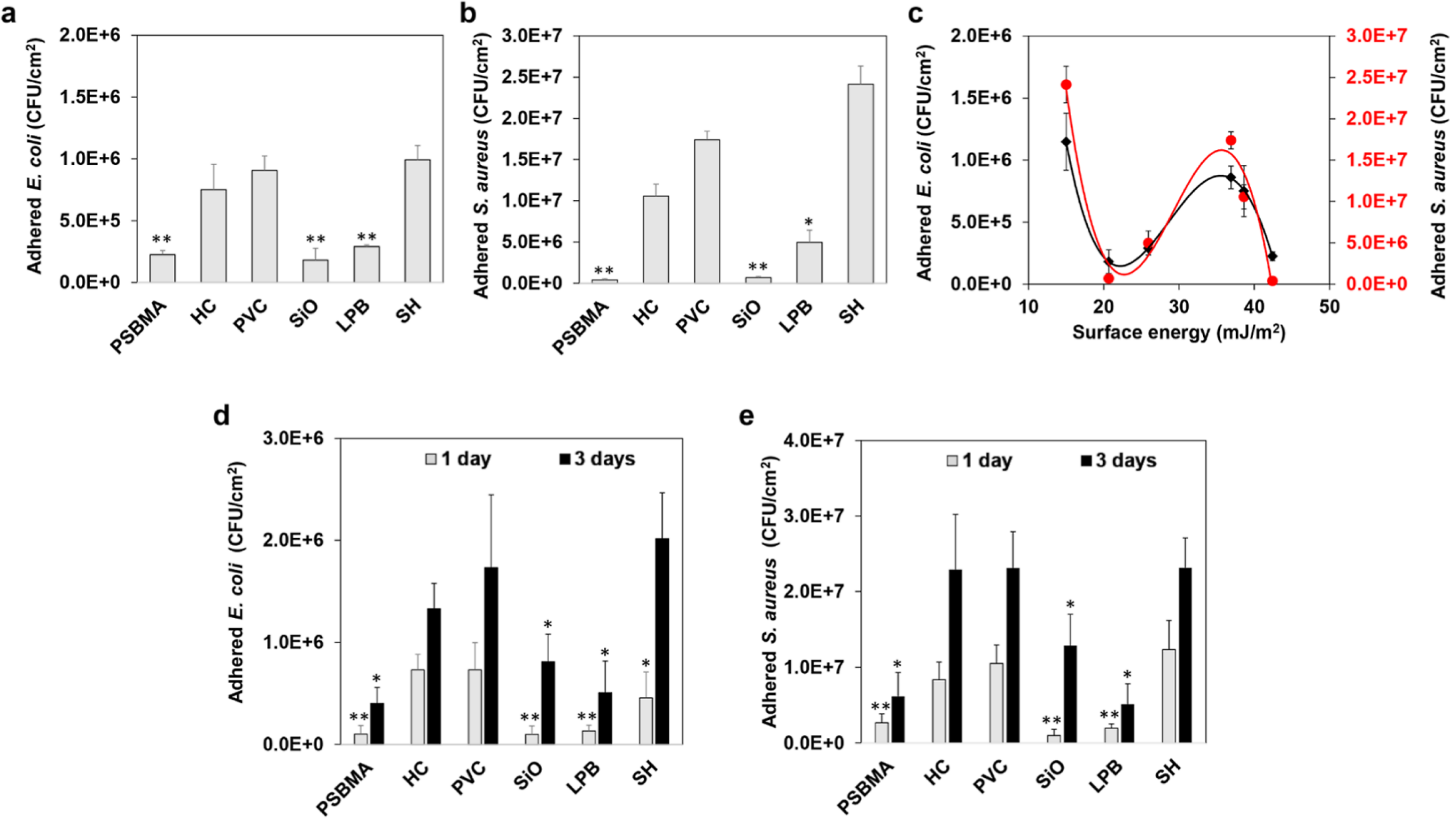
Quantitative counts of viable (a) *E. coli* and (b) *S. aureus* cells adhering to different surfaces after 24 h of static incubation; (c) the effect of surface energy on bacterial adhesion; quantitative counts of viable (d) *E. coli* and (e) *S. aureus* cells adhering to different surfaces after 24 and 72 h of dynamic incubation; (*n* = 6, bars stand for the standard deviation of the mean; *p < 0.05 and **p < 0.01 compared with PVC).

**Figure 6:**
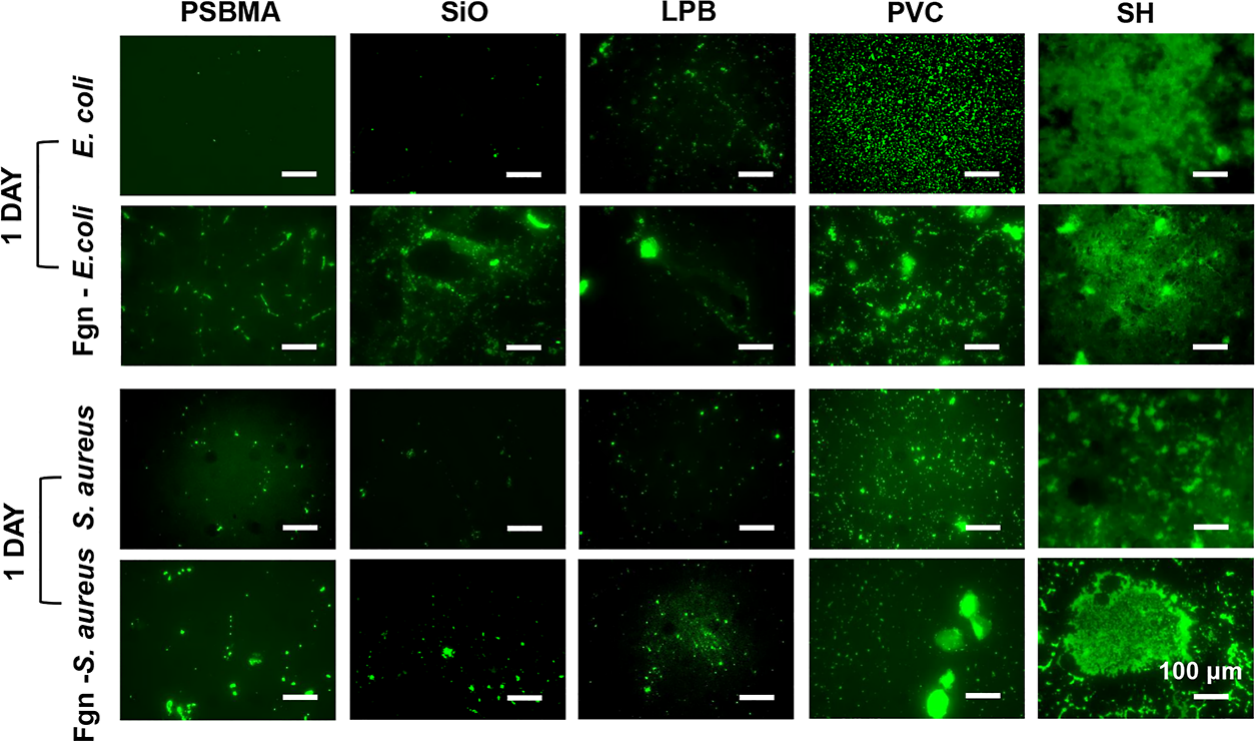
Representative fluorescent images of *E. coli* and *S. aureus* on different surfaces conditioned with neat and Fgn-supplemented PBS (scale bars correspond to 100 μm).

As Fgn deposition on catheter surfaces is not uniform, bacteria can either colonize the catheter surface or bind the accumulated Fgn to grow and form complex biofilms. We, therefore, assessed the antiadhesion performance of these surfaces against both Gram-negative and Gram-positive bacteria and investigated whether these surfaces could retain their biofilm-repelling properties after conditioning with Fgn. As a basic survival strategy, bacteria prefer to grow on a solid surface rather than in planktonic suspensions. In the absence of growth media, *S. aureus* showed a stronger binding affinity to all the surfaces than *E. coli*, as evidenced by the overall higher level of adhered cells (Figure 5). This finding was not surprising given their differences in gram-staining properties and cell wall structures.^47^ Under static conditions, the PSBMA coating and SiO-infused surface exhibited the best and most similar antiadhesion (*p* > 0.05) performance against both strains, reducing ∼75 and ∼97% of *E. coli* and *S. aureus* adhesion compared to the bare PVC (*p* < 0.01). The LPB-coated surface showed compromised repellence against both strains (*p* < 0.05), as confirmed by fluorescent microscopy (Figure 6). The results were inconsistent with an early study claiming that glass surfaces coated with SiO and LPB had equal antiadhesion efficacy.^25^ This could be ascribed to the rougher PVC substrate (Figure 1), which led to a less smooth and homogeneous brush layer with a CAH value higher than that of the SiO (Figure 2d). Under dynamic flow conditions, the SiO-infused surface, PSBMA-, and LPB-coated surfaces displayed similar antiadhesion performance (*p* > 0.05) against both strains, reducing ∼86 and ∼90% of *E. coli* and *S. aureus* adhesion compared to the bare PVC (*p* < 0.01). After 3 days, the PSBMA- and LPB-coated surfaces retained the best and similar antiadhesion activity (*p* > 0.05), while a significantly higher level of bacterial colonization was found on the SiO-infused surface (*p* < 0.05) (Figure 5d,e). As silicone oil is PDMS-based and biocompatible, it is highly unlikely that its antiadhesion activity was due to a bactericidal effect. Therefore, its compromised antiadhesion performance could be ascribed to the oil depletion in continuous flow. To verify this, we conducted a parallel experiment by measuring the oil loss from the SiO-infused samples under the same flow conditions and found that over 7.4 wt % of silicone oil was removed after 3 days (only 1.1 wt % of oil loss after 1 day). Accordingly, the water CAH increased from 1.4 to 8.7°, yielding a reduced surface repellency against bacteria. These findings indicated that the SiO-infused surface may not be feasible for long-term applications, particularly under continuous flow conditions.

Although numerous studies have reported the robust antibiofilm efficacy of SH-coated surfaces,^48^ our results suggest that their antifouling activity was poor and short-lived. After 24 h of coincubation with bacteria under both static and dynamic conditions, the SH-coated surfaces were thoroughly wetted, indicating the complete loss of air bubbles. Bacteria can enter and become trapped inside the surface structures, making it even harder to remove through gentle rinsing (Figure 6). We further attempted to correlate the initial bacterial adhesion with surface energy (Table 1) and found that there exist two separate energy regions for minimum bacterial adhesion (Figure 5c). In addition to the classic “fouling-release zone” (surface energy between 20 and 25 mJ/m^2^), the zwitterionic PSBMA with a higher surface energy also demonstrated ultralow fouling properties. This is because the neutral, watery PSBMA surface can prevent bacterial attachment by maximally reducing the electrostatic and hydrophobic attractions between bacteria and the substrate at close contact,^49^ which is different from the hydrophobic “fouling-release” surface that relies on shear flow to remove loosely (reversibly) attached bacteria.^50^

After conditioning with Fgn, an increased biomass accumulation was observed on all the surfaces, as both pathogens could express specific cell wall receptors for this host protein (Figure 6).^4^ Combined with the protein adsorption results, the PSBMA-coated surface still exhibited the best antifouling property, as only sparse and isolated cells were observed. Fgn on other surfaces triggers the aggregation of bacterial cells around the proteins. Bacteria can bind to these proteins via protein–protein interactions using EbpA and ClfB adhesins and use them as a food source to grow and produce proteases.^51,52^ Compared to bare PVC and SH, the PSBMA-coated, SiO-infused, and LPB-coated surfaces showed significantly lower levels of biomass accumulation, with no mature biofilm formed after 24 h.

**Figure 7:**
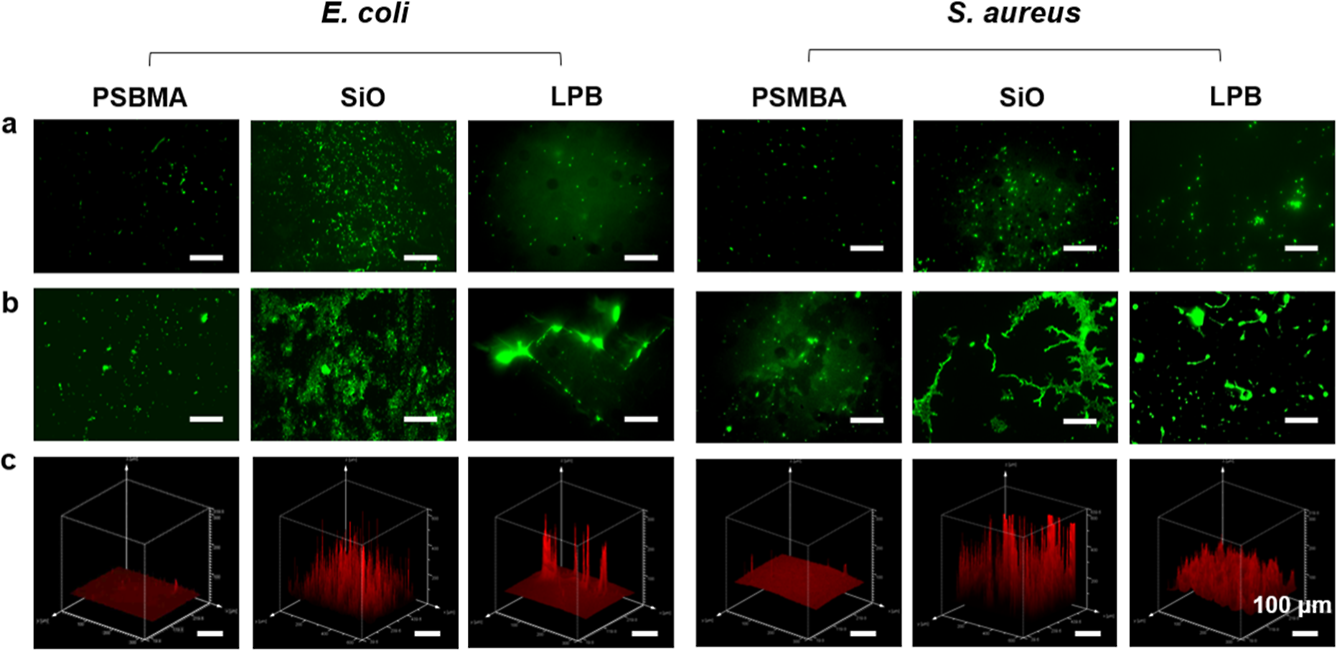
Comparison of biofilm formation on PSBMA, SiO, and LPB coatings conditioned with (a) pure PBS; (b) Fgn-supplemented PBS after 3 days; and (c) the corresponding three-dimensional images of the stained biofilm in image b (scale bars correspond to 100 μm).

Therefore, we extended the coincubation period to 3 days to assess whether these surfaces would continue to exhibit antibiofilm efficacy. As seen in Figure 7a, all of the surfaces without preconditioning with Fgn remained free of biofilm after 3 days. Compared to the PSBMA and the LPB coatings, the SiO-infused surface exhibited compromised antiadhesion activity due to oil depletion. This was further confirmed by the increased water CAH (increased from 1.4 to 9.2°) after immersing the sample in a pure PBS solution for 3 days. Instead, the covalently bound LPB demonstrated robust stability, as no significant increase in water CAH was noticed. For the Fgn-conditioned group, the PSBMA coating still showed the lowest biofouling accumulation, while biofilm in large cell aggregates and clusters was observed on the SiO-infused surface (Figure 7b). The accumulated Fgn seems to provide a framework for bacteria to grow, multiply, and develop into mature biofilm. The deposited Fgn was unevenly distributed on the LPB coating, and bacteria preferred to bind to the proteins rather than the surface, indicating that protein adsorption may be the prerequisite for biofilm formation.

### Encrustation

**Figure 8:**
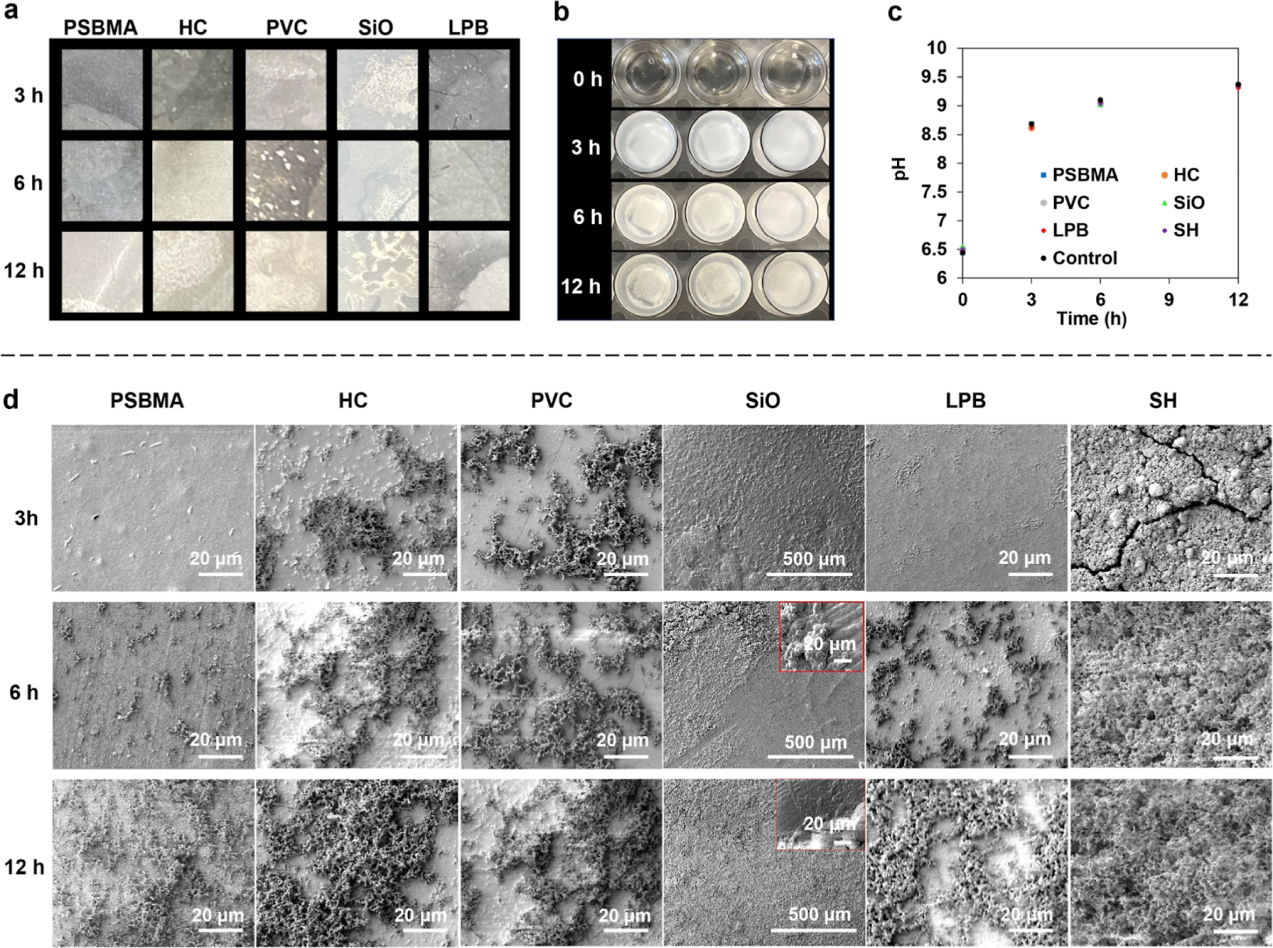
Typical images of encrustation formation (a) on different surfaces and (b) in bacterial suspensions over time; (c) pH change with time; and (d) comparison of biofouling deposited on different surfaces over time.

To assess the antiencrustation efficacy, the Fgn-conditioned samples were exposed to artificial urine with a high concentration of urea-producing bacteria (*P. mirabilis*) for up to 12 h. Bacterial adhesion and crystal deposition were observed every 3 h. As seen in Figure 8b,c, the urine pH increased from 6.5 to 8.6 within 3 h, and the urine became cloudy with crystalline deposits forming. Only sparse and isolated bacterial cells were observed on the PSBMA-, LPB-, and SH-coated surfaces, while large cell clusters were formed on the bare PVC and HC-coated surfaces. Despite no biofilm forming on the SiO-infused surface, the rough underlying PDMS substrate was visible due to oil depletion.

After 6 h, the urine pH further increased to 9.2, with more crystals accumulating in each well. The PSBMA-coated surface still exhibited the lowest biofouling adhesion, and only small and disconnected cell clusters were observed (Figure 8d). In comparison, larger cell aggregates developed on the LPB-coated surface, but the overall biofilm coverage was still lower than on other surfaces. As the antifouling performance of the LPB coating results from the dynamic nature of polymer brushes, the compromised resistance to bacterial binding is likely due to the reduced surface repellency. To verify this, we immersed the LPB-coated sample in oversaturated urine for 6 h, and the water CAH increased from 2.2 to 8.1°, indicating that the LPB coating was unable to inhibit the heterogeneous nucleation of crystals on its surface. Consequently, the compromised surface repellency results in a stronger hydrophobic–hydrophobic interaction between the surface and bacteria, accelerating bacterial binding. A similar phenomenon was observed on the SH-coated surface as the deposited crystals destroyed the air pockets, promoting bacterial colonization and biofilm formation after 3 h of immersion.

These results reveal that the watery zwitterionic coating was more effective in preventing crystal deposition than the hydrophobic LPB and SH coatings. After 12 h, all of the surfaces became fully covered with crystalline biofilm, but the encrustation was unevenly distributed (Figure 8a). As *P. mirabilis* can differentiate into hyperflagellated cells, enabling swarming migration, the attached bacteria can travel across the surface, proliferate, and form a crystalline biofilm on any random region. Despite the PSBMA and LPB coatings exhibiting improved resistance against bacterial binding and encrustation compared to other surfaces, they are unlikely to be a perfect answer to the challenges associated with urinary catheters. To provide long-term protection against CAUTI, the catheter surface should also be capable of killing adhered cells and inhibiting their migration.

## Conclusions

In this work, we successfully fabricated four types of ultralow fouling coatings for urinary catheters and investigated their potential in combating CAUTI by assessing their antibiofilm and antiencrustation properties via a stepwise approach. The superhydrophilic zwitterionic PSBMA coating demonstrated the most outstanding antifouling property, reducing over 70% of Fgn deposition and 75% of bacterial adhesion when compared to the bare PVC surface. The zwitterionic PSBMA coating also exhibited improved surface lubricity with a DCOF (0.35) close to that of HC (0.27), which may contribute to improving patient comfort and conveying additional anti-inflammatory benefits. The SiO-infused surface exhibited comparable but only short-lived antifouling activity due to its poor stability. It is noteworthy that Fgn can accumulate on all these surfaces and act as a center, facilitating bacterial binding, aggregation, and biofilm formation. Despite working through different mechanisms, both the PSBMA and LPB coatings could effectively delay biofilm formation and encrustation compared with other surfaces. Nonetheless, the colonized bacteria on their surfaces pose a challenge to their long-term antifouling efficacy, as these bacteria (e.g., *P. mirabilis*) can proliferate and migrate over the surface, ultimately leading to biofilm formation. To address this limitation, a potential strategy for future research may involve endowing these coatings with additional antibacterial and antiswarming functions.
